# Editorial: The application of new technology in the diagnosis of allergic diseases

**DOI:** 10.3389/falgy.2024.1484624

**Published:** 2024-09-25

**Authors:** Julie Weidner, Haisheng Hu, Xiangqing Hou, Baoqing Sun

**Affiliations:** ^1^ Translational Sciences and Experimental Medicine, Respiratory and Immunology (R&I), BioPharmaceuticals R&D, AstraZeneca, Mölndal, Sweden; ^2^ Department of Clinical Laboratory of the First Affiliated Hospital of Guangzhou Medical University, State Key Laboratory of Respiratory Disease, National Center for Respiratory Medicine, National Clinical Research Center for Respiratory Disease, Guangzhou Institute of Respiratory Health, Guangzhou, China; ^3^ Guangzhou National Laboratory, Bio-land, Guangzhou, China

**Keywords:** allergy, allergen detection, allergic diseases, specific immunoglobulin E (IgE), new technology

**Editorial on the Research Topic**
The application of new technology in the diagnosis of allergic diseases

## Traditional techniques for diagnosing allergic diseases

In recent years, the rising prevalence and incidence of allergic diseases have sparked heightened concerns due to their complex and poorly understood mechanisms. Effective management and prevention of it hinge largely on accurately identifying causative allergens and monitoring the risk of disease onset. Allergic diseases are mainly diagnosed on the basis of clinical symptoms, medical history, physical examination, and allergen tests (1). Traditional allergen detection techniques can be classified into two main categories: *in vivo* testing, which involves methods such as the skin prick test (SPT), intradermal test (IDT), patch test, and provocation test; and *in vitro* testing, which encompasses approaches like the serum-specific IgE (sIgE) test, total IgE (tIgE) test, and basophil activation test (BAT). All these methods are based on the patient's specific immune response to allergens to evaluate the severity of their allergy (2). With the increase of observational studies on allergen specific immunotherapy patients, we had found that some methods may not be applicable, because some patients may experience an increase in blood sIgE and sIgG4 after treatment, but the efficacy is significant (3). Therefore, with the development of technology, some new detection technologies have been applied to the diagnosis of allergic diseases, such as metabolomics, proteomics, etc. These technologies will bring a new vision to explore the mechanisms of allergic diseases and explore new biomarkers.

## Current advancements in technology for diagnosis of allergic disease

The papers in this special edition exploring the application in technology in diagnosing allergic disease, highlight the growing ambition in the field to utilize new technology to better understand the cause and consequence of allergic disease. In this collection, an in-depth overview of the interplay of allergic diseases is discussed as well as innovative ways to automate traditional tests and state-of-the-art omics based methods to further explore models of alleric disease are evaluated.

Falcon and Caoili provide a deep dive into the complex web of theories and factors involved in allergic disease and current therapeutic strategies to control allergic disease. In the past several years there has been a push to better understand and characterize the mechanism through which pharmaceuticals act in allergic disease, opening the doors for better, more effective and efficient therapies. However, more research is needed to understand the complicated relationship of genetic, environmental and immunological factors contributing to allergic diseases and how they affect the individual.

Seys et al. utilized a novel skin prick automated test (SPAT) to eliminate other variables and evaulate localization bias in the SPT. Recent studies showed that the SPT demonstrated higher sensititvity than *in vitro* molecular based tests (4), thus maintaining its status in the clinic. Despite the relative simplicity of the skin prick method variations can occur due to differences in operators and devices (5) highlighting a need for a more robust, method to reduce variability in test results. The team demonstrated that prick location did not influence the size of the wheal using the SPAT device, thus suggesting that automating the SPT may allow for a new, more consistant approach to allergy testing in the clinic.

Conjuctivitis is a common eye disease occuring in both allergic and infectious forms. Applying liquid-chromotography mass spectroscopy (LC-MS/MS) techniques, Hayashi et al. examined two models conjunctivitis in guinea pigs to examine differnces between the two disease forms. Comparison between conjunctival tissues in the two models showed infiltration of different immune cell types, with eosinophils in the case of the allergic model and neutrophils in the infectious model, thus mimicking the human disease. Conjunctival lavage fluid was analyzed for lipids by LC-MS/MS and distinct lipid profiles in the two guinea pig models. These results emphasized the different lipid mediators that are likely involved in the pathogenesis of the two disease types and identified potential biomarkers to be tested in human disease.

At present, new technologies for diagnosing allergic diseases are developing rapidly. The application of component analysis diagnostic technology reduces false positives caused by CCD interference in pollen sensitized patients, thereby more accurately diagnosing the pollen allergens that cause sensitization (6). Omics techniques have also been widely applied in risk prediction and efficacy monitoring of allergic diseases. A study conducted transcriptome deep RNA analysis on children with asthma and explore potential gene markers related to disease treatment processes, such as LINC02145, GUSBP2 and other IncRNAs (7). Besides, another study used derivatization UHPLC-Q-TOF/MS technology to explore the dynamic changes in serum metabolomics of children with asthma treated with AIT for 3 years. It was found that 12 (S)—HETE and 15 (S)—HETE may serve as biological indicators for monitoring the efficacy of AIT in children with asthma (8). In addition, there are also a study that have established an entropy change model for the biomechanics of allergic asthma patients, predicting the risk of onset of allergic asthma (7). With the emergence of more and more new technologies, the application of these technologies in the joint diagnosis of allergic diseases is worth paying attention to. However, it is difficult to explore the joint diagnostic strategies of new technologies in patients with different characteristics and scenarios, which requires further reporting in the future.
